# Barriers and Facilitators to Colposcopy Follow‐up After Abnormal Cervical Cancer Screening: Qualitative Insights From an Urban Health Care Setting

**DOI:** 10.1111/jmwh.70041

**Published:** 2025-10-29

**Authors:** Jaqueline Serrano Aguilar, Hunter K. Holt, Caroline Beshers, Kelley Baumann, Maria Valle Coto, Gelila Goba, Priyanka Gokhale

**Affiliations:** ^1^ College of Medicine University of Illinois Chicago Illinois; ^2^ Department of Family and Community Medicine University of Illinois Chicago Illinois; ^3^ College of Medicine University of Illinois Peoria Illinois; ^4^ Department of Obstetrics, Gynecology, and Reproductive Sciences Yale School of Medicine New Haven Connecticut; ^5^ Department of Obstetrics, Gynecology & Reproductive Sciences University of Miami Miller School of Medicine Miami Florida; ^6^ Department of Obstetrics and Gynecology University of Illinois Chicago Illinois

**Keywords:** abnormal Pap test, cancer prevention, cervical cancer screening, colposcopy

## Abstract

**Introduction:**

Delays to colposcopy increase the risk for cervical cancer development. Our study sought to understand the barriers and facilitators to follow‐up after an abnormal cervical cancer screening test result.

**Methods:**

English‐speaking adult patients who did not attend at least one of their scheduled appointments at an urban academic colposcopy clinic between June 2021 and June 2023 were eligible. Semistructured interviews were conducted, and thematic analyses using inductive and deductive coding were completed.

**Results:**

Twenty women were interviewed. The mean (SD) age was 34 (10) years, and participants mainly identified as non‐Hispanic Black (60%). The mean (SD) time to colposcopy was 12.5 (11.9) months. Seven participants did not have a follow‐up colposcopy at the time of the interview. Five categories of themes emerged at the individual, interpersonal, clinic, and system level, including (1) fear of pain and/or pelvic examinations, (2) patient‐provider communication (including result communication), (3) clinic interactions (including presence of trainees and lack of continuity), (4) scheduling difficulties, and (5) system‐level barriers such as loss of insurance coverage.

**Discussion:**

Barriers to follow‐up care exist across multiple levels. A one‐size‐fits‐all approach may be ineffective for facilitating follow‐up; rather, a multipronged approach may be needed to improve adherence and reduce delays to follow‐up care after an abnormal cervical cancer screening test result.

## INTRODUCTION

Cervical cancer is the fourth‐most common cause of cancer in girls and women worldwide, with more than 660,000 new cases diagnosed in 2022.^1^ Despite this, the widespread implementation of cervical cancer screening programs in the United States has led to the steady decline of US cervical cancer incidence and mortality, with an estimated 13,360 cases to be diagnosed in the United States in 2025.^2^ The recent US Food and Drug Administration approval of the self‐collected human papillomavirus (HPV) test provides new options for cervical cancer screening.^3^ Prompt follow‐up after abnormal screening result via colposcopic examination remains a critical component of diagnosis.^4^, ^5^ However, up to 44% of patients who receive an abnormal result do not undergo colposcopy.^5^, ^6^

Delays to colposcopy greater than one year have been linked to an increased risk of cancer.^4^ Although previous studies have examined barriers to care in patients with delayed follow‐up colposcopy, patients who have been missed or lost to follow‐up have not been investigated and may represent the population most at risk for cervical cancer.^4^ Additionally, the number of abnormal results requiring follow‐up will likely increase with the implementation of the newly approved self‐collected HPV test for cervical cancer screening.^7^ Therefore, the objective of this study was to better understand barriers and facilitators to care for patients who missed follow‐up after an abnormal cervical cancer screening test result.

## METHODS

English‐speaking adult patients who did not attend at least one of their follow‐up colposcopy appointments after a qualifying cervical cancer screening test result at an urban academic colposcopy clinic between June 2021 and June 2023 were eligible for the study.^8^ Our colposcopy clinic is located within a larger Center for Women's Health, where patients are seen by certified nurse midwives, nurse practitioners, and physicians. The colposcopy clinic is staffed by a rotating group of resident physicians under the supervision of an attending physician. Medical students may also have the opportunity to observe in the clinic.

**QUICK POINTS**

✦ Delays to colposcopy are linked to an increased risk of cervical cancer for patients with abnormal screening results.
✦ Barriers to completing colposcopy exist at the individual, interpersonal, clinical, and health systems levels of the patients in this study.
✦ Engaging patients with care and improving adherence to follow‐up may require a multipronged approach.

Eligible participants were abstracted from the electronic health record and were contacted via telephone by research assistants for screening, review of informed consent, and interview scheduling. Additionally, the research information and consent form were emailed to all qualifying patients for possible recruitment.

The interview guide was developed using a root cause analysis framework to introduce the topic in an open‐ended manner, followed by structured questions to assess barriers and facilitators to care.^9^ The interview questions were designed to focus on different components of the participants’ experience with (1) the last cervical cancer screening, (2) their interpretation of the results and recommended follow‐up, (3) their understanding of the procedure (colposcopy), (4) perceived barriers toward completion, and (5) ideas of what could have improved or facilitated their care (Table 1).

**Table 1:** Template for the Semistructured Interview

Topic Area	Example of Questions	Example of Probes
Open‐ended introduction	Can you tell me about your experience with cervical cancer screening?	When did you last have a Pap test? What information were you told?
Patient education and comprehension	What is your understanding of a Pap test as part of your well “woman” examination? Understanding of results and next steps? What do you know about colposcopies?	What is a Pap test? How often should it occur? What information did your doctor provide?
Barriers to follow‐up	What, if anything, kept you from following up with the recommended colposcopy?	Did you have problems with any of the following: insurance, transportation, work, child/family care? Did fear of results or pain impact the follow‐up? Did you prefer a different provider?
Process and communication	How were your results communicated? When scheduling your appointment, did you have any issues with the date offered?	What is your preferred communication method? Was communication during and after your Pap test easy to understand and follow? How well were your questions answered? How easy was it to cancel and/or reschedule if needed?
Clinic environment	How was your experience in the clinic during your Pap test?	How did the rooming process, staff, or providers impact your experience? How have you received support from family or friends during this process?
Closing remarks	Anything else you feel could have been supportive for your follow‐up?	

Interviews were conducted by 3 interviewers (J.S.A., C.B., M.V.C.), all of whom were medical students at the time of the study and had no clinical interactions with the participants. Interviewers were involved in the development of the interview framework and received training on interview best practices and techniques, including person‐centered, nonjudgmental discussions, delivered by a team member with expertise in this area (G.G.). After providing verbal consent, each participant completed a brief demographic survey. Semistructured interviews were completed via video call. All individuals contacted, regardless of their study participation, were encouraged to schedule a follow‐up visit in accordance with the guidelines and were referred to the scheduling team at the clinic.^8^ Participants received a $20 gift card by email following their interview.

**Figure 1:**
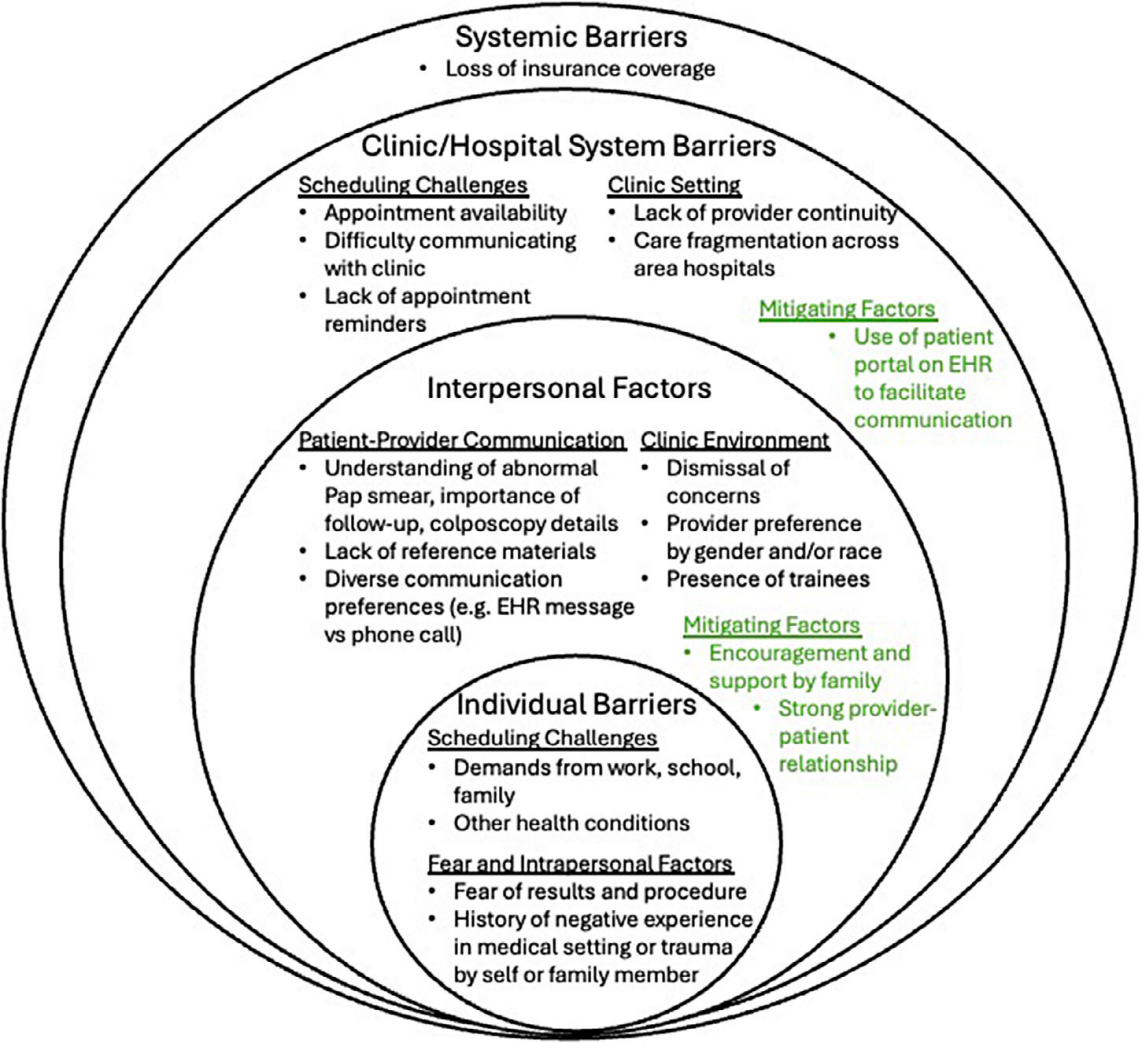
**Barriers and Mitigating Factors Using the Socioecological Framework**. This figure presents a socioecological framework of barriers to follow‐up, ranging from individual to systemic levels. Barriers include scheduling challenges, fear, communication issues, clinic dynamics, and systemic factors. Mitigating factors (in green) highlight supportive elements like patient portal use, strong provider relationships, and family encouragement Abbreviation: EHR, electronic health record.

Interview transcripts were coded by 3 of the authors using Dedoose software (Dedoose Version 9.2.007. SocioCultural Research Consultants, LLC, 2021; Los Angeles, California). The study used Braun and Clarke's reflexive thematic analysis approach; additionally, the study used both an inductive and deductive thematic analysis approach during the coding process.^10^, ^11^ Initially, the codebook was developed by reviewing and analyzing the transcripts to identify themes relating to factors that helped and/or delayed the participants’ timely follow‐up after abnormal screening result. The study team met to discuss and finalize the codebook. During coding, we used the socioecological framework to organize the findings because it helped encapsulate the nuanced interplay between barriers and mitigating factors at the individual, interpersonal, institutional, and systemic levels (Figure 1). The socioecological framework helps elucidate the complex interplay between individuals and their environments by framing the different levels of potential influence.^12^ When evaluating and analyzing codes for findings and possible themes, authors (first, second, and senior author) reflected on their positionality when evaluating their interpretation of findings as clinicians of color and their respective lived experiences. The first author was a medical student during the analysis and is now a resident in internal medicine and pediatrics. The second author is a family medicine physician and health services researcher who studies health equity in relation to cervical cancer prevention. The senior author is an obstetrician‐gynecologist who is the director of the colposcopy clinic for which this study sought to understand follow‐up and improve care for the clinic's patients.

An a priori sample size of approximately 18 to 20 participants was selected based on previous studies and systematic analyses of sample sizes in qualitative research, indicating a similar range to achieve saturation for one study site.^13^, ^14^, ^15^ The data were analyzed after completing 20 interviews and finalizing the transcription. Through initial thematic analysis of the data, we determined that an inductive thematic saturation was achieved, and recruitment was ceased.

This study was approved by the University of Illinois Chicago Institutional Review Board (STUDY2022‐1559). Findings are reported according to the Standards for Reporting Qualitative Research (Supporting Information: Appendix S1).

## RESULTS

A total of 138 individuals were eligible for participation. We recruited and interviewed 20 individuals. The mean (SD) age was 34 (10) years, and participants mainly identified as non‐Hispanic Black (60%). The mean (SD) time to colposcopy was 12.5 (11.9) months for patients who ultimately had the procedure, although 7 patients had not undergone colposcopy at the time of their interview. Most participants (n = 13) had established gynecologic care at our institution, and the remainder were referred from an outside clinic or hospital (Table 2).

**Table 2:** Participant Demographics (N = 20)

Demographic Information	Value
**Age of participants, mean (range), y**	34 (25‐55)
**Self‐identified established with gynecological care, n (%)**	
Yes	6 (30.0)
No	14 (70.0)
**Referral source, n (%)**	
Internal referral	13 (65.0)
External referral	7 (35.0)
**Education level, n (%)**	
High school	5 (25.0)
Some college	9 (45.0)
Associate's	2 (10.0)
Bachelor's	3 (15.0)
Graduate degree	1 (5.0)
**Health insurance at time of Pap test, n (%)**	
Yes	20 (100.0)
No	0 (0.0)
**Race and ethnicity, n (%)**	
Black/African American	12 (60.0)
Asian American	1 (5.0)
Hispanic/Latina	3 (15.0)
Black/African American and Native	1 (5.0)
White	2 (10.0)
Other race and/or ethnicity	1 (5.0)
**Follow‐up colposcopy completed, n (%)**	
Yes	13 (65.0)
No	7 (35.0)
**Time from abnormal Pap test result to colposcopy, mean, (SD), mo**	12.5 (11.9)

Key factors are presented using the categorical levels of the socioecological framework to help organize the relevant and related themes in the following subsections, with exemplary quotes included.

### Individual‐ and Intrapersonal‐Related Themes

Participants reported scheduling challenges at the individual level, characterized by competing demands in their lives, including work, school, and family responsibilities. Participant D acknowledged that her abnormal Pap test result gave her a “higher chance of having something”; however, she also found it difficult to balance her schedule to attend school meetings for her son and work obligations with her colposcopy appointment:

I go to school right now from 9 to 12 and then I work from 2 to 10 … So that's a lot, and then I have some meetings that I have to go to school for my son. He has a lot of meetings at school, so I put things off to deal with stuff for him and put things off for me. And I know that I shouldn't be doing that because I know that I already have a higher chance of having something because of the abnormal Pap smear. (Participant D, 22 months of delay in follow‐up)

Whether chronic or newly diagnosed, health conditions affected participants’ ability to complete the colposcopy or promptly reschedule missed appointments. Factors such as pregnancy or recent surgery further complicated follow‐up care, often leading to competing health demands. For example, Participant H struggled to complete her colposcopy after being informed of another condition that required more active surveillance:

It was just that my liver, my levels were really high. I kept going to the doctor to get lab things every week and it was still high … So that was my main concern, just lowering down that stuff. And I did have the Pap smear in mind … It was just me with the other health issues that I had. (Participant H has not had a follow‐up since an abnormal Pap smear more than one year prior to the interview)

For some participants, apprehension about pain or the possibility of abnormal results affected their follow‐up experience. Participant O expressed she was “nervous” about possible pain from to the colposcopy, although she ultimately found it to be tolerable:

That day I was a little nervous about just pain … they went more into detail about what to expect and things like that. I did get nervous about how I would tolerate the pain. I generally don't like to deal with anything painful in that area. But it was tolerable. (Participant O, 18 months of delay in follow‐up)

These feelings were at times intensified by past negative health care experiences, including feeling dismissed by providers, medical complications, or traumatic events involving family members:

They never told me that they clipped [the IUD strings]. I asked them to remove my IUD at that moment because I felt like I kept having it with BV … it was like my concerns were ignored. I knew something wasn't right because I didn't feel right. I had irregular pain that I had never had before. They were like, “oh no, everything's fine.” (Participant P has not had a follow‐up since an abnormal Pap smear more than one year prior to the interview)

Together, these experiences created psychological and emotional obstacles that made it difficult for participants to attend or reschedule their colposcopy appointments.

### Interpersonal‐Related Themes

Participants reported distinct preferences for providers based on gender and/or race, which shaped their experience in the clinic. When discussing the impact of the medical staff during the colposcopy appointment, Participant C (4 months of delay in follow‐up) “felt more uncomfortable when it's a man rather than a woman.” All clinic visits in the study setting incorporate an attending and resident with occasional participation of a medical student, which was also noted by Participant C: “there was too many people in the room. I know it's necessary, but I do not like being exposed like that to more than one person.”

Additional interpersonal barriers centered on communication between providers and patients. Participants wished for more education before undergoing the procedure. For example, Participant J (2 months of delay in follow‐up) stated that she wished she had received more “information ahead of time,” including a recommendation to “take some pain medicine beforehand to alleviate some of the pain afterwards.” Although a few participants found the patient portal helpful for accessing educational information, others noted a lack of materials they could reference later.

Communication preferences for receiving test results varied among participants. Some preferred virtual communication, particularly when results were negative. However, dissatisfaction with receiving results solely through the patient portal was a common theme. Participant C preferred a phone call from her provider, even for normal results, instead of only receiving a written message:

I know I think it was on MyChart, but I think that I wasn't happy with that because I wanted them to call me and explain, you know, over the phone, and it was just only on MyChart … I just prefer to talk to somebody over the phone. (Participant C, 4 months of delay in follow‐up)

Participants expressed feeling unsure if their pain was going to be considered, such as Participant L (2 months of delay in follow‐up), who mentioned “my pain and discomfort were gauged differently—I could be biased, but I notice a difference in the type of care that I received, and I do believe that both race and culture played a part in that.” At the same time, a few participants emphasized the positive impact of being treated by a physician of the same race. Participant L further discussed her journey to attend the colposcopy clinic within the broader context of her other interactions with the health care system: “Being a Black patient, it is important for me to have a Black doctor … I just don't believe that my concerns are noted when I have someone as a different race than what I am.”

Some patients had interpersonal facilitators that ultimately led to their follow‐up. Participant D shared her experience as she went from avoiding rescheduling the appointment due to fear and then completing the colposcopy, motivated by her young son's own emotional reaction:

I finally managed the courage to go, because when I first was told about it back in ’22 I scheduled appointments and I just got scared, and I didn't go to them until my son told me [to follow up in clinic] and he started crying, and when he got so emotional, and he's 12, I was like “oh my god, something could be wrong with me and I don't even know, and I could possibly get it taken care of, but I'm too scared to go find out, and they could probably help me.” So I managed to get the courage to finally just go and do it. Cos if you look at my history, you'll see I even missed more appointments cos I was scared. (Participant D, 22 months of delay in follow‐up)

### Clinic‐ and Hospital‐Related Themes

Participants often reported barriers related to the clinic structure, including a lack of provider continuity. For example, Participant G was unable to see her preferred provider close to home and so was seen as a new patient in the resident clinic. Similarly, Participant B (has not had a follow‐up scheduled for a Pap test at least a year ago), who was referred from an outside clinic after an abnormal Pap test result, emphasized the importance of knowing the provider's name in advance “She couldn't tell me who the physician was that was going to be doing the procedure … you need to tell me who the physician is. I need to do my due diligence.” This uncertainty was due to the rotating resident assignments, which made scheduling difficult for some patients.

Additional factors centered on communicating directly with the clinic for appointment navigation. Participant J noted she “had to wait around and if [she] missed the call,” then she would have to wait again for another phone call rather than being able to directly call to schedule or confirm her appointment. This was primarily an issue for participants who did not have the patient portal app on their phone, were referred from an outside clinic, or preferred to communicate via phone call. Similarly, a few participants were surprised to hear during our interview that they had missed a clinic appointment.

Mitigating factors included some patients’ preference to communicate online via the patient portal to facilitate communication with their health care team and reschedule appointments more easily. Participant D (22 months of delay in follow‐up) reported receiving effective appointment reminders: “They did everything—contacting me and bugging me like they weren't gonna leave me alone about it … I appreciated it because they're more concerned than I am about my health.” Participants’ ability to access the patient portal or use their preferred communication methods influenced the timeliness and completion of their follow‐up care.

### Health Care System–Related Themes

Participants were surveyed about whether systemic issues, such as access to childcare or transportation issues, impacted their ability to follow up after an abnormal Pap test result (Table 1). Moreover, all participants were assessed for changes or lapses in their health insurance coverage. The majority reported no change in their health insurance status from the time of their Pap test to the interview; however, this was not true for all patients. After Participant H initially rescheduled her colposcopy appointment, her insurance coverage lapsed, preventing her from completing the procedure again. During our interview, she mentioned that she could not pay out of pocket because she remained uninsured and without follow‐up:

I called again around middle October to make another appointment for November and they give it to me, but they give it to me for December and I had already lost my insurance on the 30th … I was supposed to go but … that's when my insurance got cancelled. And honestly I don't have money to be paying out of pocket right now for the services or anything like that, so I haven't followed up with them. (Participant H has not had a follow‐up since abnormal Pap smear more than one year prior to the interview)

## DISCUSSION

Our results highlight the complex interplay of multiple factors at different levels that influence a patient's ability to follow up after an abnormal cervical cancer screening result. Participants described barriers ranging from individual to systems‐based. These barriers often compounded one another, leading to prolonged delays in care. However, participants also identified mitigating factors that supported their follow‐up. Understanding these barriers and facilitators through a socioecological framework is essential for designing more effective interventions. Addressing follow‐up care after an abnormal cervical cancer screening result requires a multifaceted approach that enhances patient education and communication strategies while also improving workflows in health care settings to reduce logistical obstacles and build trust between patients and providers.^16^

A key goal of our study was to explore potential interventions to address the barriers and mitigating factors identified in the interviews with participants who missed appointments. Given the varied preferences reported, ranging from appointment reminder texts and electronic health record–based rescheduling to direct phone calls with a health care team member for questions and concerns, it is essential to offer flexible options. Prior research has shown that even when multiple communication methods are available, patients may be unaware of these options or find that their preferences are not implemented.^17^ Regularly surveying patients during registration about their preferred communication method and integrating these preferences into clinic workflows could improve follow‐up adherence and patient engagement. Additionally, recognizing that many patients prefer direct communication with their provider is crucial. Relying solely on the electronic health record and text messages may feel impersonal and could negatively impact adherence.

Themes were identified at the interpersonal level, such as patient education, comprehension, and interactions within the clinic environment, including participants identifying or having their concerns acknowledged by their provider. Addressing knowledge gaps is particularly important given the risk of lesion progression with delayed follow‐up.^4^ Some participants expressed fear of developing cervical cancer after an abnormal screening result but hesitated to schedule their follow‐up appointment until prompted by family members or after receiving multiple reminders from the clinic.

Understanding how patients seek and access health care information is crucial for creating effective educational resources. Kulkarni et al conducted a qualitative study on barriers to colposcopy, including questions to evaluate internet use and its connection to appointment adherence.^13^ Their findings suggest that written materials were not commonly used, with most patients relying on online resources for information. Before implementing an effective intervention, it is important to assess the quality and accessibility of online information from trusted sources to ensure patients have reliable materials for reference.

Additionally, many participants noted that a strong physician‐patient relationship or support from other health care providers had a positive influence on their medical care and adherence to follow‐up appointments. Conversely, limited knowledge about the etiology and progression of cervical cancer, as well as the indications for colposcopy, emerged as a common theme, possibly due to gaps in patient education or difficulty obtaining answers to their follow‐up questions after their visit.

Since the completion of this study, our clinic has developed a formal system to connect with patients in advance of their appointment. Previously, patients were referred for colposcopy and scheduled by a customer service representative. All appointment reminders were automated. We now have a nurse practitioner who contacts patients in advance of their appointment to review scheduling and provide an overview of the procedure. Additionally, we have implemented a formal outreach system to connect with patients who have missed colposcopy appointments via phone and the patient portal in a standardized manner. Further work will be needed to evaluate the effectiveness of these interventions on patient attendance.

Particular attention should be given to our study's demographic composition, which highlights our clinic's predominantly Black patient population in an urban academic center that serves mainly publicly insured patients. We also acknowledge that this study has multiple limitations. The opinions and concerns of the cohort of eligible patients willing to participate in such a study may not fully align with those of patients who may not feel comfortable with study participation. Another factor to consider is the trust established between the participant and researcher during the interview, which was shaped by the limitations of a virtual setting. Conducting multiple interviews may have helped strengthen this rapport. Additionally, some participants shared nuanced experiences with the health care system, including stigma and pain perception, although these themes were not explored in depth. Finally, this study was conducted at a single institution with a specific system and workflow, as well as a specific patient population, in a major metropolitan area. These findings may not be applicable to all clinical settings.

## CONFLICT OF INTEREST

The authors have no conflicts of interest to disclose.

## Supporting information




**Appendix S1**. Standards for Reporting Qualitative Research (SRQR) Checklist

## References

[1] Bray F , Laversanne M , Sung H , et al. Global cancer statistics 2022: GLOBOCAN estimates of incidence and mortality worldwide for 36 cancers in 185 countries. CA Cancer J Clin. 2024;74(3):229‐263. doi:10.3322/caac.21834 38572751

[2] Cancer Stat Facts: cervical cancer . National Cancer Institute website. Accessed August 11, 2025. https://seer.cancer.gov/statfacts/html/cervix.html.

[3] FDA Roundup: May 17, 2024. US Food & Drug Administration website. Accessed August 18, 2025. https://www.fda.gov/news‐events/press‐announcements/fda‐roundup‐may‐17‐2024.

[4] Alimena S , Lykken JM , Tiro JA , et al. Timing of colposcopy and risk of cervical cancer. Obstet Gynecol. 2023;142(5):1125‐1134. doi:10.1097/aog.0000000000005313 37607530 PMC10637756

[5] Chao CR , Chubak J , Beaber EF , et al. Gaps in the screening process for women diagnosed with cervical cancer in four diverse US health care settings. Cancer Med. 2023;12(3):3705‐3717. doi:10.1002/cam4.5226 36106421 PMC9939213

[6] Liang LA , Zeissig SR , Schauberger G , et al. Colposcopy non‐attendance following an abnormal cervical cancer screening result: a prospective population‐based cohort study. BMC Womens Health. 2022;22(1):285. doi:10.1186/s12905-022-01851-6 35810270 PMC9270801

[7] Montealegre JR , Hilsenbeck SG , Bulsara S , et al. Self‐collection for cervical cancer screening in a safety‐net setting: the PRESTIS randomized clinical trial. JAMA Intern Med. 2025;185(9):1119‐1127. doi:10.1001/jamainternmed.2025.2971 40478588 PMC12144659

[8] Perkins RB , Guido RS , Castle PE , et al. 2019 ASCCP risk‐based management consensus guidelines for abnormal cervical cancer screening tests and cancer precursors. J Low Genit Tract Dis. 2020;24(2):102‐131. doi:10.1097/lgt.0000000000000525 32243307 PMC7147428

[9] Sharma AE , Lyson HC , Cherian R , Somsouk M , Schillinger D , Sarkar U . A root cause analysis of barriers to timely colonoscopy in California safety‐net health systems. J Patient Saf. 2022;18(1):e163‐e171. doi:10.1097/pts.0000000000000718 32467445 PMC7688501

[10] Braun V , Clarke V . Using thematic analysis in psychology. Qual Res Psychol. 2006;3(2):77‐101. doi:10.1191/1478088706qp063oa

[11] Braun V , Clarke V . Thematic Analysis: A Practical Guide. SAGE Publications; 2021.

[12] Golden SD , Earp JAL . Social ecological approaches to individuals and their contexts:twenty years of health education & behavior health promotion interventions. Health Educ Behav. 2012;39(3):364‐372. doi:10.1177/1090198111418634 22267868

[13] Kulkarni A , Glynn S , Gamble CR , et al. Understanding perceived barriers to colposcopy follow‐up among underserved women at an urban teaching hospital: a qualitative study. J Low Genit Tract Dis. 2023;27(1):87‐92. doi:10.1097/lgt.0000000000000700 36074132

[14] Hennink M , Kaiser BN . Sample sizes for saturation in qualitative research: a systematic review of empirical tests. Soc Sci Med. 2022;292:114523. doi:10.1016/j.socscimed.2021.114523 34785096

[15] Vasileiou K , Barnett J , Thorpe S , Young T . Characterising and justifying sample size sufficiency in interview‐based studies: systematic analysis of qualitative health research over a 15‐year period. BMC Med Res Method. 2018;18(1):148. doi:10.1186/s12874-018-0594-70 PMC6249736 30463515

[16] Wordlaw‐Stinson L , Jones S , Little S , et al. Challenges and recommendations to recruiting women who do not adhere to follow‐up gynecological care. Open J Prev Med. 2014;4(3):123‐128. doi:10.4236/ojpm.2014.43017 24991485 PMC4075769

[17] Alkomos MF , Mendez D , Mazzei‐Pifano D , et al. Patients’ reasons for missing scheduled clinic appointments and their solutions at a major urban‐based academic medical center. J Community Hosp Intern Med Perspect. 2020;10(5):426‐430. doi:10.1080/20009666.2020.1796903 33235676 PMC7671744

